# Supplementary material for: Frequency stabilization and noise-induced spectral narrowing in resonators with zero dispersion
Source: Nat Commun. 2019 Sep 2;10:3930. doi: 10.1038/s41467-019-11946-8 (PMC6718662; doi:10.1038/s41467-019-11946-8)
Supplement: Supplementary file 1 — Supplementary Information [file 41467_2019_11946_MOESM1_ESM.pdf]

Supplementary Information

Frequency stabilization and noise-induced spectral narrowing in resonators with zero dispersion

Huang *et al.*

Supplementary Note 1. Theory of frequency of eigenoscillation vs. its amplitude and theoretical calculation of resonance response curves

Eigenoscillation is defined as an oscillation in an idealized conservative system. It involves theoretical approximation that neglects dissipative and time-dependent forces. In relation to the system defined by Eqs. (1)-(4), it means that we should neglect in the equation of motion (1) the friction $-2\Gamma\dot{q}$ and average an explicit dependence of other forces on time, if any. The spring restoring force does not explicitly depend on time and therefore the averaging has no effects on it. The electric force may explicitly depend on time and thereby, for V in Eq. (4), we replace V^2 in Eq. (3) for the electric force by its average over time $\overline{V^2}$:

$$\overline{V^2} = V_{\mathrm{dc}}^2 + V_{\mathrm{ac}}^2/2 + \langle V_{\mathrm{n}}^2 \rangle + V_{\mathrm{c}}^2/2 \qquad (1)$$

The non-resonant (high-frequency) ac voltage $V_{\mathrm{c1}}(t) \equiv V_{\mathrm{c}}\cos(\omega_{\mathrm{c}}t)$ is present in all our experiments, so that $V_{\mathrm{c}}^2/2$ is to be kept in $\overline{V^2}$ in all cases. As for $V_{\mathrm{ac}}^2/2$, it is either exactly equal to zero (in the experiments on fluctuation spectra) or being much smaller than $V_{\mathrm{c}}^2/2$. Therefore we neglect it in all cases. Finally, in those experiments where noise is present, the average of its square $\langle V_{\mathrm{n}}^2 \rangle$ is much smaller than V_{dc}^2 (and even $V_{\mathrm{c}}^2/2$). Nevertheless we do take it into account in $\overline{V^2}$ in order to further improve the agreement with the experiment (see Supplementary Note 3 and Figs. 3a and 3b in the main text). In the present Supplementary Note however, we assume for the sake of simplicity of notations that noise is absent or negligible i.e.

$$\overline{V^2} = \tilde{V}_{\mathrm{dc}}^2 \equiv V_{\mathrm{dc}}^2 + V_{\mathrm{c}}^2/2. \qquad (2)$$

Next, we discuss the calculation of the frequency of eigenoscillation ω vs. its amplitude a, assuming that $\tilde{V}_{\mathrm{dc}}^2$ has a given value from the range defined in Eq. (6) while

a is sufficiently small for the deviation of $\omega(a)$ from its zero-amplitude limit $\omega(a \to 0) \equiv \omega_1$ to be small i.e. $|\omega(a) - \omega_1| \ll \omega_1$. After the omission of friction in the equation of motion and the replacement of V^2 by $\overline{V^2} = \tilde{V}_{\rm dc}^2$, we transform from q to $x = q - q_{\rm eq}$ where $q_{\rm eq} \approx \tilde{v}g$ is the equilibrium position, expand the force into the Taylor series in powers of x and, allowing for the smallness of $\tilde{v}$ in the relevant range of $\tilde{V}_{\rm dc}^2$, neglect in coefficients of the Taylor series terms proportional to $\tilde{v}^n$ with $n \geq 2$. As can be shown (cf. [1, 2]), it is sufficient to keep in the Taylor series only terms up to the 5th power if we are interested only in the quartic approximation for $\omega(a)$ (see Eq. (11)) while the latter, in turn, is sufficient for our case. So, we end up with the following equation of motion:

$$\ddot{x} = -\sum_{n=1}^{5} \alpha_n x^n, \tag{3}$$

where α_n are given in Eqs. (8)-(10) of the main text and in the paragraph following Eq. (10). Next, we apply to this system the method of successive approximations described in Refs. [[1, 2]]. Namely, we seek the solution of the equation of motion in the form of series $\sum_{i=1}^{\infty} x^{(i)}$ where $x^{(i)}$ with a given i is proportional to a^i while

$$x^{(1)} = a\cos(\omega t), \tag{4}$$

where $\omega \equiv \omega(a)$ is the exact frequency of eigenoscillation the first harmonic of which has the amplitude a. In turn, $\omega(a)$ is sought in the form of series $\sum_{i=0}^{\infty} \omega^{(i)}$ where $\omega^{(i)}$ with a given i is proportional to a^i while

$$\omega^{(0)} = \sqrt{\alpha_1} . \tag{5}$$

The coefficient $\omega^{(0)}$ (Supplementary Equation 5) immediately gives us the formula for ω_1 in the approximation of $\omega(a)$ by Eq. (11). Allowing for Eq. (8) for α_1, we obtain from it Eq. (12).

In order to find the next-order terms in the series for x and ω, i.e. $x^{(2)}$ and $\omega^{(1)}$, we first present the equation of motion (Supplementary Equation 3) in the following equivalent form

$$\frac{\left(\omega^{(0)}\right)^2}{\omega^2}\ddot{x} + \left(\omega^{(0)}\right)^2 x = -\sum_{n=2}^{5}\alpha_n x^n - \left(1 - \frac{\left(\omega^{(0)}\right)^2}{\omega^2}\right)\ddot{x}\ , \qquad (6)$$

then substitute into it the series for x, and neglect in both sides of the equation small terms proportional to a^i with $i \geq 3$. Terms proportional to a cancel each other owing to the proper choice of $x^{(1)}$ and $\omega^{(0)}$, so that we end up with the closed equation for terms of the second order in a. Using the identity $(\cos(\omega t))^2 = (1 + \cos(2\omega t))/2$, we present this equation as follows:

$$\ddot{x}^{(2)} + \left(\omega^{(0)}\right)^2 x^{(2)} = -\frac{\alpha_2 a^2}{2} - \frac{\alpha_2 a^2}{2}\cos(2\omega t) - 2a\omega^{(0)}\omega^{(1)}\cos(\omega t)\ . \qquad (7)$$

The above equation has a simple interpretation: it is an equation of motion of the linear (harmonic) oscillator with the eigenfrequency $\omega^{(0)}$ (left-hand side of the equation) driven by forces of two distinctly different types in the present context. The first type is represented by the first and second terms on the right-hand side: they are distinctly non-resonant. The second type is represented by the last term on the right-hand side: it is almost resonant. But the constrained vibrations under action of an almost resonant force would have a large amplitude, which would contradict to the original assumption that an amplitude of each successive correction to the solution is small. In order to avoid such a contradiction, the value of $\omega^{(1)}$ should be chosen in such a way that the amplitude of the "almost resonant force" would turn into zero. The latter takes place only if we impose the condition

$$\omega^{(1)} = 0. \qquad (8)$$

This value of $\omega^{(1)}$ provides the deletion of the almost resonant force from Supplementary Equation 7. Solving the inhomogeneous linear differential equation (Supplementary Equation 7) with the remaining inhomogeneous part in a usual way, we have

$$x^{(2)} = -\frac{\alpha_2 a^2}{2\left(\omega^{(0)}\right)^2} + \frac{\alpha_2 a^2}{6\left(\omega^{(0)}\right)^2}\cos(2\omega t). \tag{9}$$

In order to find the next-order terms in the series for x and ω, i.e. $x^{(3)}$ and $\omega^{(2)}$, we perform the analysis analogously to the previous step: substitute the series in the equation of motion (Supplementary Equation 6) and neglect terms which are proportional to a^i with $i \geq 4$ while terms proportional to a cancel each other owing to the proper choice of $x^{(1)}$ and $\omega^{(0)}$ and terms proportional to a^2 cancel each other owing to the proper choice of $x^{(2)}$ and $\omega^{(1)}$, so that we end up with the closed equation for terms of the third order in a. Similar to the previous step, the resulting differential equation for $x^{(3)}$ represents the equation of motion of the linear oscillator driven both by a distinctly non-resonant force and by an almost resonant one:

$$\ddot{x}^{(3)} + \left(\omega^{(0)}\right)^2 x^{(3)} = -a^3\left(\frac{\alpha_3}{4} + \frac{\alpha_2^2}{6\left(\omega^{(0)}\right)^2}\right)\cos(3\omega t) + a\left(2\omega^{(0)}\omega^{(2)} + \frac{5a^2\,\alpha_2^2}{6\left(\omega^{(0)}\right)^2} - \frac{3a^2\alpha_3}{4}\right)\cos(\omega t). \tag{10}$$

Then we again apply one of the key ideas of the method: we find $\omega^{(2)}$ from the condition that the amplitude of the almost resonant force should turn into zero. The result is the following:

$$\omega^{(2)} = a^2\left(\frac{3\alpha_3}{8\omega^{(0)}} - \frac{5\alpha_2^2}{12\left(\omega^{(0)}\right)^3}\right). \tag{11}$$

Solving then the resulting equation (Supplementary Equation 10) in a usual manner, we easily find $x^{(3)}$.

One can continue such iterative process to any order. It may be worth noting however that, as the order of an approximation further increases, calculations become very cumbersome: even the ultimate expressions are quite unwieldy. Thus, $\omega^{(4)}$ is given by the following formula (note that $\omega^{(3)}$, like any other odd-order correction, is equal to zero):

$$\omega^{(4)} = a^4 \frac{1}{16\,\omega^{(0)}}\left(5\alpha_5 - \frac{15\alpha_3^2}{16\left(\omega^{(0)}\right)^2} - \frac{14\alpha_2\alpha_4}{\left(\omega^{(0)}\right)^2} + \frac{22\alpha_3\alpha_2^2}{3\left(\omega^{(0)}\right)^4} - \frac{49\alpha_2^4}{36\left(\omega^{(0)}\right)^6}\right). \qquad (12)$$

In our case however, the situation greatly simplifies due to the smallness of the parameter $\tilde{\nu}$. In particular, the even coefficients α_{2i} are proportional to $\tilde{\nu}$ and it is seen from Supplementary Equations 11 and 12 that all terms which include one or more even coefficients α_{2i} with $i \geq 1$ are proportional to $\tilde{\nu}^n$ with $n \geq 2$ and therefore may be neglected in comparison with other terms.

Consider first Supplementary Equation 11 for $\omega^{(2)}$. Dividing it by a^2, neglecting terms including even coefficients α_{2i}, allowing for the formula (Supplementary Equation 5) for $\omega^{(0)}$ (together with the first-order approximation (8) for α_1) and using the first-order approximation (9) for α_3, we obtain Eq. (13) for the coefficient κ in the quadratic term of the quartic approximation (11) for $\omega(a)$.

The situation with Supplementary Equation 12 is more subtle. Dividing it by a^4, neglecting terms including the even coefficients (α_2 or α_4), using the zero-order approximation (8) for α_1 and first-order approximations (9) and (10) for α_3 and α_5 respectively, and taking into account the definition of the nonlinearity length scale $L_\mathrm{n} \equiv$

$\omega_s/\sqrt{\beta_s}$, we obtain the following expression for the coefficient η in the quartic term of the quartic approximation (11) for $\omega(a)$:

$$\eta = -\frac{15\omega_s\tilde{\nu}}{8g^4}\left\{1 - \frac{8\mu_s L_n^2}{3\beta_s}\frac{[g/(2L_n)]^2}{\tilde{\nu}}\left(\frac{g}{2L_n}\right)^2 + \frac{1}{2}\tilde{\nu}\left(\frac{[g/(2L_n)]^2}{\tilde{\nu}} - 1\right)^2\right\}. \qquad (13)$$

The third term in the curly parentheses is $\gtrsim \tilde{\nu}$ in the relevant range indicated in Eq. (6), where $\tilde{\nu}/[g/(2L_n)]^2 \sim 1$, and therefore it can be neglected as compared to the first term 1. Furthermore, characteristic scales of spring nonlinearities of close orders are typically close to each other, in particular this concerns nonlinearities of the third and fifth orders and, therefore, $\mu_s L_n^2/\beta_s \sim 1$. Hence, for the relevant range indicated in Eq. (6), the second term in the parentheses is of the order of $[g/(2L_n)]^2 \ll 1$ so that it can also be neglected. Thus, Supplementary Equation 13 for η reduces to Eq. (14), meaning that η is strongly dominated by the electrostatic contribution and is distinctly negative, which nicely conforms to the experiment.

As for κ, the ratio of the contributions from the electric and spring forces is represented by the second term in the parentheses of Eq. (13). This term is a ratio of two small parameters. The most interesting (in the zero-dispersion context) part of the range of the dc voltage indicated in Eq. (6) corresponds to the case when $\tilde{\nu}$ is barely exceeded by $[g/(2L_n)]^2$ (in other words, when the spring contribution weakly dominates over the electrostatic one), that yields a small but positive κ.

Since κ is positive while η is negative, $\omega(a)$ (11) possesses a local maximum and, moreover, as the excess of 1 over the ratio $\tilde{\nu}/[g/(2L_n)]^2$ is small, the maximum lies in the range of a being much smaller than g, where the quartic approximation (11) adequately describes the true $\omega(a)$, that proves the self-consistency of the theory.

If $\tilde{\nu}$ is much smaller than $[g/(2L_\mathrm{n})]^2$ (i.e. if $\tilde{V}_\mathrm{dc}^2 \ll V_\mathrm{zd}^2$), then the above arguments in favor of the domination of the electrostatic contribution over the spring contribution are no longer valid and, in fact, it is vice versa for sufficiently small values of $\tilde{\nu}$: the spring contribution dominates i.e. $\eta \approx \eta_\mathrm{s}$, thus being positive. However it does not mean that the resonator does not possess the zero-dispersion property in this case i.e. that $\omega(a)$ does not possess a local maximum: it does, which can be shown by a different method (which will be presented by us elsewhere), but the maximum occurs at much larger values of a, namely the relevant range lies close to g and the quartic approximation (11) is evidently insufficient for an adequate description of $\omega(a)$.

Thus we conclude that the value $V_\mathrm{zd}^2 \equiv \frac{\beta_\mathrm{s} m g^5}{2\varepsilon S}$, corresponding to the zero value of κ, represents the boundary between zero-dispersion and conventional nonlinear regimes in terms of $\tilde{V}_\mathrm{dc}^2$. It may be worth noting that it does not depend on the elasticity of the springs i.e. on the Hooke's coefficient.

Consider now the resonator subject to a weak resonant ac driving $F_\mathrm{ac}\cos(\omega_\mathrm{d} t)$. The theoretical approximation of the resonance curves $A(\omega_\mathrm{d})$ is determined by the equation[1, 2]

$$4m^2\omega_1^2 A^2\{[\omega(a = A) - \omega_\mathrm{d}]^2 + \Gamma^2\} = F_\mathrm{ac}^2, \quad (14)$$

where the eigenoscillation amplitude a in $\omega(a)$ is replaced with the amplitude of constrained vibrations A.

In experiments, $\omega(a{=}A)$ is measured by recording the resonance curves at many values of the driving amplitude. By identifying the peak of the curves, we obtain the approximate backbone line $A_\mathrm{peak}(\omega_\mathrm{peak})$[1]. For any given value of A_peak, the reversed function $\omega_\mathrm{peak}(A_\mathrm{peak})$ is approximately equal to the frequency of eigenoscillation with the

amplitude equal to the given A_{peak}, i.e. $\omega(a) \approx \omega_{\text{peak}}(A_{\text{peak}} = a)$. The transformed backbone line (e.g. in Fig. 2b and Fig. 2d) of ω_{peak} vs. A_{peak}^2 well approximates the eigenfrequency vs. the scaled energy:

$$\omega(E) \approx \omega(a^2 = E/\{m\omega_1^2/2\}) \approx \omega_{\text{peak}}(A_{\text{peak}}^2 = E/\{m\omega_1^2/2\}). \qquad (15)$$

Furthermore, the measured transformed backbone line is well fitted by the parabolic function both in the zero-dispersion and conventional regimes (Figs. 2b and 2d respectively), that allows us to find the corresponding coefficients κ and η in the approximation of $\omega(a)$ (11). This approximation of $\omega(a)$ was substituted then in Supplementary Equation 14 in order to find the theoretical approximations of the resonance curves $A(\omega_d)$ for given values of the dc voltage V_{dc} and the driving amplitude F_{ac}: see thin solid lines in Figs. 2a and 2c. The agreement with the experimental lines is satisfactory.

<u>Supplementary Note 2. Effect of the quadratic noise and the white-noise approximation.</u>

There are just few requirements to the form of the voltage noise component $V_{\text{n}}(t)$ which are crucial for the experiment: its spectrum (spectral power density) should represent a narrow peak with the maximum close to ω_1 and the width greatly exceeding the width of the frequency range relevant to the fluctuation spectrum peaks in a vicinity of ω_1. On the other hand the width of the V_{n} spectrum peak should be sufficiently small for the quadratic noise to be negligible (otherwise such noise may smear zero-dispersion effects). For the sake of brevity and clarity, we consider below only the form of the noise which

was used in our experiments (still keeping some extent of generality where possible) while a generalization is straightforward.

V_n used in the experiments can be presented in the following form:

$$V_\mathrm{n}(t) = N_\mathrm{c}(t)\cos(\omega_1 t) + N_\mathrm{s}(t)\sin(\omega_1 t), \tag{16}$$

where $N_\mathrm{c}(t)$ and $N_\mathrm{s}(t)$ are identical independent noise sources with the zero average and a monotonously decaying correlation function,

$$\langle N_\mathrm{c}(t)\rangle = \langle N_\mathrm{s}(t)\rangle = 0, \tag{17}$$

$$\langle N_\mathrm{c}(t) N_\mathrm{s}(t')\rangle = \langle N_\mathrm{s}(t) N_\mathrm{c}(t')\rangle = 0, \tag{18}$$

$$\langle N_\mathrm{c}(t) N_\mathrm{c}(t')\rangle = \langle N_\mathrm{s}(t) N_\mathrm{s}(t')\rangle = 2I_\mathrm{n} d(t-t'), \tag{19}$$

where $t - t' \geq 0$ and $d(\tau)$ is a monotonously and slowly (as compared to the period of eigenoscillation $2\pi/\omega_1$) decaying function normalized similarly to the δ–function:

$$\int_0^\infty \mathrm{d}\tau d(\tau) = \frac{1}{2} . \tag{20}$$

Using Supplementary Equations 16 to 19, we can easily derive formulas both for the average and for the correlation function of V_n :

$$\langle V_\mathrm{n}(t)\rangle = 0, \qquad \langle V_\mathrm{n}(t) V_\mathrm{n}(t')\rangle = 2I_\mathrm{n} d(t-t')\cos\left[\omega_1 (t-t')\right]. \tag{21}$$

It may be expected that $d(\tau)$ is characterized with a single decay time-scale $(2d(0))^{-1}$ which may also be interpreted as a correlation time-scale $t_\mathrm{cor} \equiv (2d(0))^{-1}$. Measurements confirm that $V_\mathrm{n}(t)$ in our experiment satisfies the above requirement. Indeed, the smoothed spectrum has a Gaussian form (Supplementary Figure 1):

$$Q^{(V_n)}(\Omega) \equiv \frac{1}{\pi}\mathrm{Re}\left[\int_0^\infty \mathrm{d}t \left\langle V_n(t)V_n(0)\right\rangle \exp(\text{-i}\Omega t)\right] \approx \frac{I_n}{2\pi}\exp\left\{-\frac{1}{2}\left(\frac{\Omega-\omega_1}{\pi f_{bd}/\sqrt{2\ln(2)}}\right)^2\right\}, \qquad (22)$$

and therefore $d(\tau)$ is Gaussian as well, namely:

$$d(\tau) = \frac{1}{2t_{cor}}\exp\left\{-\frac{\pi}{4}\left(\frac{\tau}{t_{cor}}\right)^2\right\}, \qquad t_{cor} \equiv \frac{\sqrt{\ln(2)/\pi}}{f_{bd}} \ . \qquad (23)$$

The noise intensity I_n, which may also be interpreted as a product of 2π and the maximum of the noise power spectrum $Q_{max}^{(V_n)} \approx Q^{(V_n)}(\omega_1)$ (Supplementary Figure 1), is small in the sense that $\left\langle N_c^2\right\rangle = \left\langle N_s^2\right\rangle \equiv \left\langle N^2\right\rangle << V_{dc}^2$ so that the quadratic noise plays a negligible role. Allowing for Supplementary Equation 19 and the identity $t_{cor} \equiv (2d(0))^{-1}$, this strong inequality is equivalent to a satisfaction of the following condition:

$$\lambda \equiv \frac{I_n}{t_{cor}V_{dc}^2} << 1 \ . \qquad (24)$$

As concerns t_{cor}, on the one hand, it greatly exceeds the period of natural oscillation, that allows us in particular to satisfy the condition (Supplementary Equation 24) in the relevant range of noise intensity but, on the other hand, it is much smaller than a reciprocal of the characteristic half-width of any of distinct spectral peaks in the spectrum of fluctuations of the system (1)-(4) (for $V_d \equiv 0$). As it is seen from the consideration below, such smallness of t_{cor} allows us to consider the noise in the context of the fluctuation spectrum as white.

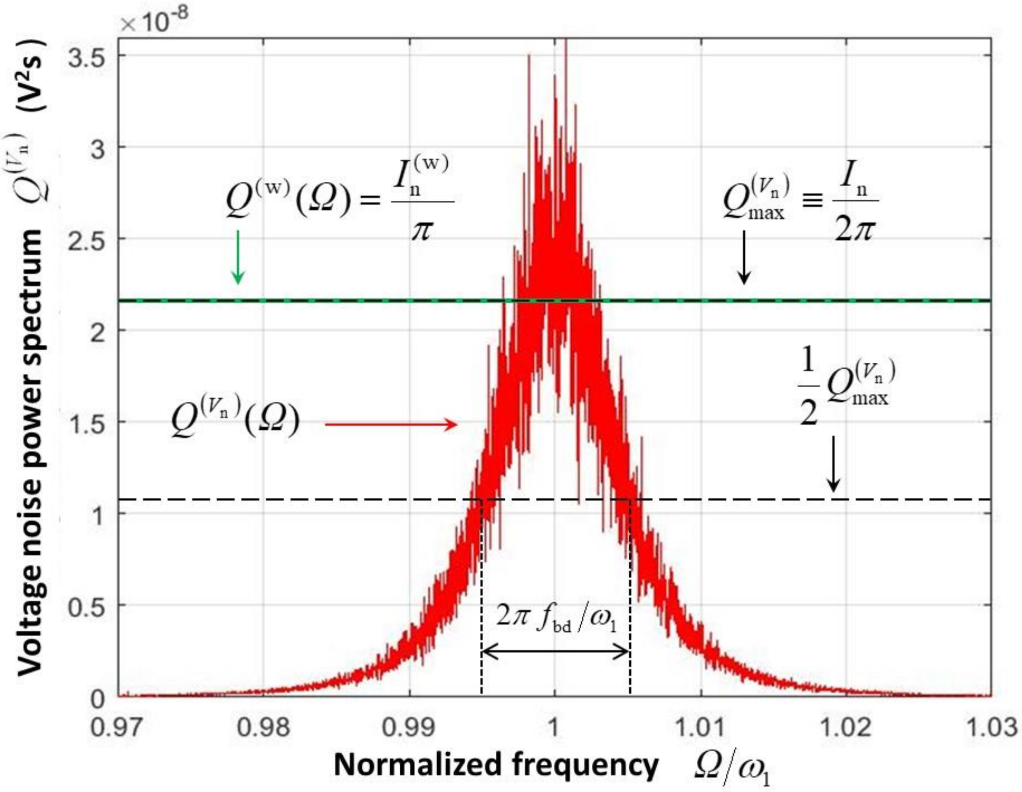

Supplementary Figure 1 An example of the voltage noise power spectrum. Example of the power spectrum of noise component of voltage $Q^{(V_n)}(\Omega)$ (jagged solid red line): this particular noise acted on the resonator in the conventional regime ($V_{dc} = -2.3$ V) where the noise intensity $D_w = 4470$ pN^2 Hz^{-1} (see Supplementary Equation 38 below). Fluctuations are due to a limited number of data. Maximum of the smoothed over fluctuations spectrum and its half-level are indicated by the corresponding labels with arrows and by the horizontal dashed lines. The intersection of the lower of the dashed lines with the smoothed spectrum defines the range of the "bandwidth" of the spectrum. The higher dashed line (indicating the maximum) coincides with the thick solid green line showing the white noise spectrum $Q^{(w)}(\Omega)$ of the intensity twice smaller than that of the original noise: its action on the system is almost the same as that by the original noise.

Let us substitute V [Eq.(4)] with $V_{\mathrm{d}} \equiv 0$ and V_{n} (Supplementary Equation 16) into the equation of motion given by Eqs. (1)-(3), neglect the high-frequency terms (similar to the analysis of the ac-driving), keep only those of noise-induced terms which have the lowest order of the smallness parameter λ (the latter procedure includes in particular neglecting the "quadratic" noise term proportional to $\left(N_{\mathrm{c}}^2+N_{\mathrm{s}}^2\right)/2-\langle N^2\rangle$, the intensity of which is of the order of λ as compared to that of the linear noise term F_{n}/m explicated below), and take into account the smallness of the zero-dispersion energy $E_{\mathrm{zd}} \equiv m\omega_1^2 q_{\mathrm{zd}}^2/2$ as compared to the characteristic energy $m\omega_1^2 g^2/2$ (which allows us to neglect a deviation of $(1-q/g)^2$ from unity in the correlation function of the linear noise term F_{n}/m). The result can be presented in the following form:

$$\ddot{q} = -2\Gamma\dot{q} + \frac{1}{m}\left\{F_{\mathrm{s}}(q) + F_{\mathrm{e}}\left(q, V^2 \to \tilde{V}_{\mathrm{dc}}^2 + \langle N^2\rangle\right)\right\} + \frac{1}{m}F_{\mathrm{n}}, \tag{25}$$

where the expression in the curly parentheses may be considered as a generalized potential force $\mathrm{d}U^{(\mathrm{n})}(q)/\mathrm{d}q$ which just slightly differs from that in the absence of noise,

$$\begin{aligned} U^{(\mathrm{n})}(q) &\equiv U^{(\mathrm{n})}(q,\lambda) \approx U_{\mathrm{s}}(q) + U_{\mathrm{e}}(q)(1+\lambda) \quad, \\ U_{\mathrm{s}}(q) &\equiv \frac{1}{2}m\omega_{\mathrm{s}}^2 q^2 + \frac{1}{4}m\beta_{\mathrm{s}} q^4 + \frac{1}{6}m\mu_{\mathrm{s}} q^6 + \ldots, \\ U_{\mathrm{e}}(q) &\equiv -\frac{\varepsilon S\tilde{V}_{\mathrm{dc}}^2}{2(g-q)}, \end{aligned} \tag{26}$$

and F_{n}, for which we introduce the notion "linear noise force", reads as

$$F_{\mathrm{n}} \equiv F_{\mathrm{e}}\left(q, V^2 \to 2V_{\mathrm{dc}}V_{\mathrm{n}}\right) = -\frac{\varepsilon S V_{\mathrm{dc}}}{g^2}V_{\mathrm{n}} \ . \tag{27}$$

We will show in the rest of Supplementary Note 2 that, if we replace in the simplified equation of motion (Supplementary Equation 25) the linear noise force F_{n}

(Supplementary Equation 27), which possesses distinct oscillatory properties (see the definition of V_{n} in Supplementary Equations 16 to 20), by the white noise specified in the main text (and below), most important features of dynamics of the system (Supplementary Equation 25) paradoxically remain almost unaffected, provided the system does not go in the phase space too far beyond a vicinity of the stable state of the resonator in the absence of noise. In particular, this relates to the dynamics determining the spectrum of fluctuations.

In intuitive terms, this paradox is explained as follows. The oscillator is almost linear and therefore the involved range of its eigenfrequencies is very narrow for relevant values of noise intensity, which is why its reaction to external forces has a strongly frequency-selective nature i.e. sharply weakens as a frequency of an external force goes beyond the aforementioned relevant narrow range of eigenfrequencies. Despite the spectrum of the original noise (Supplementary Equation 27) is narrow in comparison with its central frequency, it nevertheless fully covers the yet more narrow relevant range of eigenfrequencies and, moreover, includes also relatively broad ranges below and above the latter. That is why an addition to its spectrum of components of a comparable magnitude which lie even farther from the relevant range of eigenfrequencies cannot significantly affect the system. This in particular concerns the strictly white noise of such intensity at which the spectrum of the white noise approximately coincides with the spectrum of the original narrow-band noise (Supplementary Equation 27) in the relevant range of eigenfrequencies of the system i.e. in the close vicinity to ω_1, which approximately corresponds to the maximum of the spectrum.

Let us present a rigorous substantiation of this important property. To this end, we transform in the equation of motion (Supplementary Equation 25) to energy and slow angle[3] (one could use other slow variables, e.g. those exploited in [4], too but we choose these since the angle is immediately related via $\omega(E)$ to the spectrum of involved eigenoscillations). More concretely, we first present a single 2nd-order differential equation of motion as the equivalent system of two 1st-order differential equations for q and $p \equiv \dot{q}$, transform to energy E and angle φ[2, 3], and take into account that the nonlinearity is weak which is why the dependence of q and p on E and φ may be approximated by that for the harmonic oscillator, i.e.

$$q - q_{\mathrm{eq}}^{(\mathrm{n})} \approx \frac{\sqrt{2E/m}}{\omega_1}\cos(\varphi), \qquad p \approx \sqrt{2mE}\sin(\varphi) \tag{28}$$

($q_{\mathrm{eq}}^{(\mathrm{n})}$ is the equilibrium (local minimum) position for $U^{(\mathrm{n})}(q)$ (Supplementary Equation 26): it slightly differs from q_{eq}).

Then we transform from the angle to the slow angle,

$$\tilde{\varphi} = \varphi - \omega_1 t \ , \tag{29}$$

and neglect in the resulting equation fast-oscillating terms, in accordance with the averaging method[1]. Ultimately, we obtain the following stochastic equations for energy and slow angle:

$$\dot{E} = -2\Gamma E - N_E(t),$$
$$\dot{\tilde{\varphi}} = \omega(E) - \omega_1 - N_{\tilde{\varphi}}(t), \tag{30}$$

where N_E and $N_{\tilde{\varphi}}$ denote the following parametric noise terms:

$$
\begin{aligned}
N_E(t) &= \frac{\varepsilon S V_{\mathrm{dc}}}{g^2}\left(\frac{2E}{m}\right)^{\frac{1}{2}} \frac{\sin(\tilde{\varphi})N_{\mathrm{c}}(t)+\cos(\tilde{\varphi})N_{\mathrm{s}}(t)}{2}, \\
N_{\tilde{\varphi}}(t) &= \frac{\varepsilon S V_{\mathrm{dc}}}{g^2}(2mE)^{-\frac{1}{2}} \frac{\cos(\tilde{\varphi})N_{\mathrm{c}}(t)-\sin(\tilde{\varphi})N_{\mathrm{s}}(t)}{2}.
\end{aligned}
\tag{31}
$$

The deterministic part of the dynamics of the system (Supplementary Equations 30 to 31) is slow in the sense that characteristic time-scales at which E and $\tilde{\varphi}$ significantly change greatly exceed the correlation time of noise t_{cor} (Supplementary Equation 23). Therefore, $d(t-t')$ in (Supplementary Equation 19) may be approximated by the delta-function in the context of main features of the dynamics (Supplementary Equations 30 to 31) (in particular, this concerns the spectrum of fluctuations):

$$
d(t-t') \approx \delta(t-t'). \tag{32}
$$

Since pair correlators of noise terms in the dynamic equations (Supplementary Equations 30 to 31) are δ-correlated in the approximation (Supplementary Equation 32), the non-stationary probability density $W(E,\tilde{\varphi},t)$ obeys the Fokker-Planck equation (FPE)[5],

$$
\frac{\partial W}{\partial t} = \hat{L}_{\mathrm{FP}} W , \tag{33}
$$

where the Fokker Planck operator $\hat{L}_{\mathrm{FP}}$ can be presented in the form

$$
\hat{L}_{\mathrm{FP}} \equiv -\frac{\partial}{\partial E}D_E - \frac{\partial}{\partial \tilde{\varphi}}D_{\tilde{\varphi}} + \frac{\partial^2}{\partial E^2}D_{EE} + \frac{\partial^2}{\partial E \partial \tilde{\varphi}}\left(D_{E\tilde{\varphi}} + D_{\tilde{\varphi}E}\right) + \frac{\partial^2}{\partial \tilde{\varphi}^2}D_{\tilde{\varphi}\tilde{\varphi}}, \tag{34}
$$

where the drift and diffusion coefficients are fully determined by the form of the deterministic terms in the equation of motion (Supplementary Equations 30 to 31) and by the multipliers of δ-functions in the pair correlators of the corresponding stochastic terms. Applying the general formulas[5] to the stochastic system (Supplementary Equations 30 to 31) with noises $N_{\mathrm{c}}(t)$ and $N_{\mathrm{s}}(t)$ given by Supplementary Equations 17 to 19 where

$d(t-t')$ is approximated by the δ-function, we obtain the following expressions for the drift and diffusion coefficients:

$$D_E = -2\Gamma E + \frac{D}{2m}, \qquad D_{\tilde{\varphi}} = \omega(E) - \omega_1,$$
$$D_{EE} = \frac{DE}{2m}, \qquad D_{E\tilde{\varphi}} = D_{\tilde{\varphi}E} = 0, \qquad D_{\tilde{\varphi}\tilde{\varphi}} = \frac{D}{8mE}, \tag{35}$$

where D is an intensity of the linear noise force F_{n} :

$$\langle F_{\mathrm{n}}(t) F_{\mathrm{n}}(t') \rangle = 2Dd(t-t')\cos[\omega_1(t-t')], \qquad D \equiv \left(\frac{\varepsilon S V_{\mathrm{dc}}}{g^2}\right)^2 I_{\mathrm{n}} \quad . \tag{36}$$

Let us introduce the strictly white (i.e. strictly δ-correlated) noise $V_{\mathrm{n}}^{(\mathrm{w})}$ the intensity of which is twice smaller than the intensity of the original noise V_{n} given by Supplementary Equation 16:

$$\langle V_{\mathrm{n}}^{(\mathrm{w})}(t) V_{\mathrm{n}}^{(\mathrm{w})}(t') \rangle = 2I_{\mathrm{n}}^{(\mathrm{w})}\delta(t-t'), \qquad I_{\mathrm{n}}^{(\mathrm{w})} = \frac{I_{\mathrm{n}}}{2} \ . \tag{37}$$

Let us now replace the original noise V_{n} in Supplementary Equation 27 for F_{n} by $V_{\mathrm{n}}^{(\mathrm{w})}$. In terms of the linear noise force, it means that the real force F_{n} in the equation of motion given by Supplementary Equation 25 is replaced by the white noise $F_{\mathrm{n}}^{(\mathrm{w})}$ of the twice smaller intensity:

$$\langle F_{\mathrm{n}}^{(\mathrm{w})}(t) F_{\mathrm{n}}^{(\mathrm{w})}(t') \rangle = 2D^{(\mathrm{w})}\delta(t-t'), \qquad D_{\mathrm{w}} = \frac{D}{2} \equiv \left(\frac{\varepsilon S V_{\mathrm{dc}}}{g^2}\right)^2 \frac{I_{\mathrm{n}}}{2} \ . \tag{38}$$

Let us first, similarly to the case of the real noise (Supplementary Equation 16), transform in the equation of motion (Supplementary Equation 25) to energy and slow angle. The averaging over fast oscillations in the system is a more subtle issue as compared to the case of the noise (Supplementary Equation 16). In the latter case, the correlation time of noise

greatly exceeds the period of fast oscillations and therefore the averaging may be applied immediately to the stochastic terms within the equation of motion. In contrast, the correlation time of the strictly white noise is zero and therefore the period of "fast" oscillations is slow as compared to it. Thus, the averaging of stochastic terms would be invalid. In order to correctly eliminate fast oscillations within the stochastic motion, we need first to transform to the description within the FPE while the averaging over fast oscillations should be done afterwards: the FPE is a deterministic equation and difficulties related to stochastic equations do not arise here. After the averaging is carried out in such a way, the resulting averaged FPE exactly coincides with the FPE relevant for stochastic equations being a result of the averaging of the equations of motion (Supplementary Equations 30 to 31), i.e. the drift and diffusion coefficients coincide after the averaging with those given in Supplementary Equation 35. It follows from this that main statistical properties of the two systems almost coincide: a relative difference is of the order of a ratio of period of eigenoscillation to the correlation time i.e. $2\pi/(\omega_1 t_{\rm cor}) << 1$ (in particular, this concerns the spectrum of fluctuations). At the same time, it is worth noting that the equation of motion (Supplementary Equation 25) itself is a result of a neglect by small terms proportional to D and an accurate estimate of some of them requires a more sophisticated white-noise approximation than that of the main term, but we do not take into account these small terms in the present work.

We emphasize that the spectrum of the idealized (white) voltage noise $V_{\rm n}^{(\rm w)}$ given in Supplementary Equation 37 coincides with the maximum value of that of the real voltage noise (Supplementary Equations 16 to 20) (Supplementary Figure 1). It is the most general requirement for a white noise to adequately mimic an original narrow-band noise: this

requirement does not depend either on a concrete general form of the original noise or on a concrete shape of a correlation function $d(t)$ of its slow component.

Supplementary Note 3. Theoretical calculation of the spectrum of fluctuations.

The spectrum of fluctuations of a given dynamical variable is commonly defined as the half-Fourier transform of a correlation function of this variable [3-7],

$$\tilde{Q}(\omega) \equiv \frac{1}{\pi} \mathrm{Re}\left[\int_0^\infty \mathrm{d}t Q(t) \exp(-\mathrm{i}\omega t) \right]. \tag{39}$$

and, often, a relevant dynamical variable is a generalized coordinate[3, 4, 7, 8] q. So, let us explicate the definition of the coordinate correlation function $Q(t)$. To the best of our knowledge, the most general definition existing in the literature is the following[3, 8]:

$$Q(t) = \lim_{\tau_1 \to \infty} \frac{1}{2\tau_1} \int_{-\tau_1}^{\tau_1} \mathrm{d}\tau \left\langle \left(q(\tau + t) - \langle q(\tau + t) \rangle \right) \left(q(\tau) - \langle q(\tau) \rangle \right) \right\rangle, \tag{40}$$

where $\langle q(\xi) \rangle$ is a value of q averaged over a statistical distribution of the system states at a given instant ξ while the outer brackets $\langle \ldots \rangle$ mean an averaging over a statistical distribution of the system states both at the "initial" instant τ and at the "final" instant $\tau + t$. Consider a conditional probability density $\tilde{W}(q, p, \tau + t | q_0, p_0, \tau)$: the notation means that, if the system has coordinate q_0 and momentum p_0 at a given instant τ , then the probability for the coordinate and momentum of the system at a given later instant $\tau + t$ to lie within the infinitesimally narrow intervals $[q, q + \mathrm{d}q]$ and $[p, p + \mathrm{dp}]$ respectively is equal to $\tilde{W}(q, p, \tau + t | q_0, p_0, \tau) \mathrm{d}q \mathrm{d}p$. There are classes of systems where, as time t increases, $\tilde{W}$ approaches a stationary distribution independent of the initial

conditions: $\tilde{W}_{\text{st}}(q,p) \equiv \lim_{t\to\infty}\left[\tilde{W}(q,p,\tau+t|q_0,p_0,\tau)\right]$. In such a case, the expression (Supplementary Equation 40) for the correlation function can be presented in a more explicit form:

$$Q(t) = \int_{-\infty}^{\infty}\int_{-\infty}^{\infty}\int_{-\infty}^{\infty}\int_{-\infty}^{\infty} \mathrm{d}q\mathrm{d}p\mathrm{d}q_0\mathrm{d}p_0 \tilde{W}_{\text{st}}(q_0,p_0)\tilde{W}(q,p,t|q_0,p_0,0)\left[(q-\langle q\rangle)(q_0-\langle q\rangle)\right], \quad (41)$$

where $\langle q\rangle$ is the value of q averaged over the stationary distribution,

$$\langle q\rangle \equiv \int_{-\infty}^{\infty}\int_{-\infty}^{\infty} \mathrm{d}q\mathrm{d}p\tilde{W}_{\text{st}}(q,p)q\,. \quad (42)$$

The broadest and most important class of systems possessing a stationary distribution is formed by systems moving in a potential field which goes to infinity as the coordinate goes to plus or minus infinity while being subject to a linear friction and an additive white noise with a constant intensity[5]:

$$\dot{p} = -2\Gamma p - \frac{dU(q)}{dq} + f_{\text{n}}(t),$$
$$\dot{q} = \text{p}, \quad (43)$$

where

$$\langle f_{\text{n}}(t)\rangle = 0, \qquad \langle f_{\text{n}}(t)f_{\text{n}}(t')\rangle = 4mk_{\text{B}}T\Gamma\delta(t-t'), \quad (44)$$

where k_{B} is the Boltzmann constant while T has a meaning of an effective temperature.

Such a system possesses the Gibbsian stationary distribution:

$$\tilde{W}_{\text{st}}(q,p) = \tilde{W}_{\text{Gibbs}}(E) \equiv \frac{1}{Z}\exp\left(-\frac{E}{k_{\text{B}}T}\right),$$
$$E \equiv E(q,p) = U(q) + \frac{p^2}{2m}, \quad (45)$$
$$Z = \left\{\iint \mathrm{d}q\mathrm{d}p\exp\left(-\frac{E}{k_{\text{B}}T}\right)\right\}^{-1}.$$

Before proceeding to our case, we need to generalize the definition of the fluctuation spectrum. Let a system of interest be metastable [5, 9] rather than truly stable. For the sake of simplicity, let us restrict the further discussion to potential systems i.e. those of the type (Supplementary Equation 43) but a potential field $U(q)$ may be arbitrary and noise $f_n(t)$ may not necessarily be white. A potential of a system possessing a metastable state necessarily includes at least one barrier so that, if the system exceeds the barrier, then it escapes from the metastable state for a long time or even forever. If the system is subject to noise, then the energy required for the escape is gained by the system from noise. If noise intensity is sufficiently small, then the mean escape time t_{esc} [3, 5, 8-14], i.e. the average time during which the optimal (large) fluctuation [3, 10-13] required for the escape occurs, is exponentially (activation-like) large as compared both to a time-scale t_{qs} during which a quasi-stationary distribution is established in the vicinity of the metastable state and to a time-scale t_{cd} at which a correlation of motion in the vicinity of the metastable state substantially decays. Therefore, if to replace the upper integration limit in the definition of the spectrum (Supplementary Equation 39) by a large time t_l from the range limited by the strong inequalities $\max\{t_{qs}, t_{cd}\} \ll t_l$ and $t_l \ll t_{esc}$ while doing a statistical averaging in $Q(t)$ (Supplementary Equation 40) over a quasi-stationary distribution, then the integral

$$\tilde{Q}(\omega) \equiv \frac{1}{\pi} \mathrm{Re}\left[\int_0^{t_l} \mathrm{d}t Q(t) \exp(-\mathrm{i}\omega t)\right] \qquad (\max\{t_{qs}, t_{cd}\} << t_l << t_{esc}) \qquad (46)$$

characterizes fluctuations in the vicinity of the metastable state analogously to how $\tilde{Q}(\omega)$ (Supplementary Equation 39) characterizes fluctuations in the vicinity of a stable state.

The spectrum measured by us in this work should be understood just in the way described in the previous paragraph since our resonator in the relevant range of $\tilde{V}_{\mathrm{dc}}^2$ is a metastable system for which t_{esc} is very large if the noise intensity lies in the relevant range indicated in Eq. (6). Let us demonstrate this explicitly. The phase plane of the noise-free system described by Eqs. (1)-(3) with $V^2 = \tilde{V}_{\mathrm{dc}}^2$ (while $\tilde{V}_{\mathrm{dc}}^2$ lies within the range indicated in Eq. (6)) possesses both a stable and unstable stationary states: respectively ($q = q_{\mathrm{eq}} \approx \tilde{\nu} g, \dot{q} = 0$), which corresponds to the bottom of the corresponding potential well of $U^{(\mathrm{n})}(q, \lambda = 0) \equiv U(q) = U_{\mathrm{s}}(q) + U_{\mathrm{e}}(q)$ (Supplementary Equation 26), and ($q = q_{\mathrm{b}}, \dot{q} = 0$), which corresponds to the top of the barrier of $U(q)$ situated at

$$q_{\mathrm{b}} \approx g\left(1 - \sqrt{\tilde{\nu}}\right). \tag{47}$$

The height of the barrier is:

$$\Delta U \equiv U(q_{\mathrm{b}}) - U(q_{\mathrm{eq}}) \approx \frac{m\omega_{\mathrm{s}}^2 g^2}{2}. \tag{48}$$

As q increases beyond q_{b}, the potential $U(q)$ sharply decreases and ultimately drops to $-\infty$ as q approaches g: if the top plate gets into such a close vicinity of the electrode, then it most likely sticks to it soon after that.

As soon as noise (e.g. random force $f_{\mathrm{n}}(t)$ in Supplementary Equation 43) is added, the originally stable state in the bottom of the potential well becomes metastable since there is a non-zero probability P for the large fluctuation which transfers the resonator over the barrier to occur. If noise is weak, then the dependence of this probability on the noise intensity D_{w} is activation-like (cf. [3, 5, 8-14]):

$$P \propto \exp\left(-\frac{S}{D_{\text{w}}}\right), \tag{49}$$

where S is called action. Consider for example the system (Supplementary Equation 43) where the noise force $f_{\text{n}}(t)$ may generally speaking be arbitrary. If the correlation time of noise is much smaller than the period of natural oscillations $2\pi/\omega_1$ and the noise is additive (independent of q and p, apart from being linear) i.e. if $f_{\text{n}}(t)$ is well approximated by Supplementary Equation 44, then action is equal to $2m\Gamma\Delta U$ so that the absolute value of the exponent in Supplementary Equation 49 is equal to $\Delta U/k_{\text{B}}T$ (cf. [3, 9, 10, 12]). However neither of the two above conditions is satisfied. On the one hand, the noise in our original equation of motion given by Eqs. (1)-(4) can be considered as independent of q only in the vicinity of the metastable state, where the multiplier $(1-q/g)^{-1}$ can be approximated by 1, while the latter approximation is invalid in the major part of the energy range relevant to the escape i.e. where $E-U\left(q_{\text{eq}}\right)\sim\Delta U$: owing to this q-dependence, the effective noise intensity grows as energy increases (and even diverges as $E-U\left(q_{\text{eq}}\right)$ approaches ΔU), that leads to the decrease of action and therefore to the exponentially large increase of P (Supplementary Equation 49). If noise correlation time is much smaller than the period of natural oscillation $2\pi/\omega_1$, the exponent in P (Supplementary Equation 49) could be shown to be smaller of the conventional value $\Delta U/k_{\text{B}}T$ by the factor $7/2$. But the correlation time is, on the contrary, much larger than $2\pi/\omega_1$. Moreover, given that the eigenfrequency $\omega(E)$ goes to zero as energy E approaches $U(q_{\text{b}})$, our noise (the spectrum of which concentrates

near the frequency ω_1) is strongly non-resonant with eigenoscillations in the energy range close to the barrier value $U(q_b)$. Previous theoretical analysis (see e.g. [3, 10, 12,]) showed that the optimal fluctuation which yields the conventional exponent $-\Delta U/k_B T$ in P (Supplementary Equation 49) for the system (Supplementary Equations 43 to 44) is equal to $f_n^{(opt)}(t) = 2\Gamma p$: it results in the most probable escape path being time-reversed to the noise-free relaxational path from the top of the potential barrier into the bottom of the potential well [3, 10, 12]. Given that $\Gamma/\omega_1 << 1$, this relaxational path represents almost ideal eigenoscillations with the frequency $\omega(E(t))$ while $E(t)$ slowly relaxes from $U(q_b)$ to $U(q_{eq})$. Therefore the aforementioned optimal fluctuation oscillates in the energy range close to $U(q_b)$ with the slowly decreasing frequency $\omega(E(t)) << \omega_1$ while $E(t)$ slowly (with a characteristic time-scale $\omega_1/(\Gamma\omega(E(t)))$) increases within this range as time goes. Unlike the white noise (Supplementary Equation 44), the spectrum of the noise in our experiment does not contain so small frequencies and therefore such realization merely does not exist for it. Obviously, those realizations which do exist yield much larger action. This effect is much stronger than the aforementioned tendency to an increase of action owing to the q-dependence of the noise force. That is why, altogether S / D_w greatly exceeds $\Delta U/k_B T$ and, as a result, the escape probability is vanishingly small provided the ratio $\Delta U/k_B T$ is large (or even moderate). Such conclusion conforms to our experiments: we did not observe any sticking of the plates when the noise intensity was within the range at which the noise-induced spectral narrowing took place.

Thus, either experimental or theoretical calculation of the fluctuation spectrum for our resonator in the relevant range of noise intensity does not differ in practice from cases

of strictly stable systems. For example, while doing theoretical calculation, we may formally replace the unstable part of the potential function $U(q)$ (i.e. that for $q > q_{\mathrm{b}}$) by any growing function: results for the spectrum near the natural frequency are not sensitive to such a replacement to an exponential accuracy.

As it is shown in Supplementary Note 2, the stochastic dynamics of the resonator in the presence of a resonant narrow-band noise component of voltage, e.g. of the form in Supplementary Equations 16 to 20, is well described within the additive linear noise approximation (Supplementary Equation 25) and, moreover, if the real noise F_{n} is replaced by the white noise $F_{\mathrm{n}}^{(\mathrm{w})}$ of a twice smaller intensity as explicitly defined in Supplementary Equation 38, main statistical features of the dynamics in the vicinity of the stable state (just such features determine the fluctuation spectrum in the range of the natural frequency of the resonator) remain almost the same. Allowing for this and for the above discussion of the equivalence to an exponential accuracy between the quasi-stationary distribution in the metastable system and the stationary distribution in the auxiliary stable system, we conclude that the resonator driven by the noise (Supplementary Equations 16 to 20) possesses a quasi-steady distribution close to the Gibbsian stationary distribution [3, 5-7]:

$$\begin{aligned}
&\tilde{W}_{\mathrm{st}}(q,p) = \tilde{W}_{\mathrm{Gibbs}}(E) \equiv \frac{1}{Z}\exp\left(-\frac{E}{D_{\mathrm{w}}/(2m\Gamma)}\right),\\
&E \equiv E(q,p) = U^{(\mathrm{n})}(q) + \frac{p^2}{2m} \approx \frac{m\omega_1^2 q^2}{2} + \frac{p^2}{2m},\\
&Z = \left\{\iint \mathrm{d}q\mathrm{d}p \exp\left(-\frac{E}{D_{\mathrm{w}}/(2m\Gamma)}\right)\right\}^{-1} \approx \frac{4\pi\Gamma}{\omega_1 D_{\mathrm{w}}},
\end{aligned} \tag{50}$$

where D_{w} is defined in Supplementary Equation 38 in Supplementary Note 2.

One more remarkable property of systems with the white noise is that the time evolution of the non-stationary probability density $\widetilde{W}$ obeys the Fokker-Planck equation [3-5, 7] (FPE) i.e certain partial differential equation of the second order (cf. Supplementary Equations 33 to 35 in Supplementary Note 2). Still, its solution is complicated, even in case of a weak nonlinearity and even numerically. The latter is especially true in our weakly nonlinear case, where pronounced characteristic features of the fluctuation spectrum may result from rather subtle differences of an evolution of the probability density from that in the purely linear case and from that in a conventional weakly nonlinear case. Fortunately, a powerful method for the FPE solution and the calculation of fluctuation spectra on its base was developed for underdamped cases earlier: first for monostable potentials[7] and then for multistable ones[15]. As a result, a solution of the complicated partial differential equation and a heavy integration over a double phase space and time is reduced to a solution of a relatively simple ordinary differential equation with boundary conditions and a simple integration just over energy. One can find details in the aforementioned papers[7, 15] or in the review[3]. Here, we just refer to the relevant general result, presenting it in notations used in (or just relevant to) the present paper. The spectrum of fluctuations reads as follows:

$$\tilde{Q}(\Omega) \approx 2\,\mathrm{Re}\left[\int_0^{E_{\mathrm{up}}} \mathrm{d}E\, \frac{1}{\omega^{(\mathrm{n})}(E)} \left(q_1^{(\mathrm{n})}(E)\right)^* W_1(E,\Omega)\right], \qquad (51)$$
$$\left|\Omega - \omega_1\right| << \omega_1,$$

where the superscript $\ldots^{(\mathrm{n})}$ here and thereafter means a modification of a given quantity owing to the slight modification of the original potential by the quadratic noise (see $U^{(\mathrm{n})}(q)$ Supplementary Equation 26), E_{up} is a rather arbitrarily chosen energy which

greatly exceeds an average energy in the quasistationary state for a given noise intensity $D_{\rm w}$ but being lower than the potential barrier height $\Delta U^{(\rm n)}$:

$$\begin{aligned} &\langle E\rangle << E_{\rm up} < \Delta U^{(\rm n)}, \\ &\langle E\rangle \approx \frac{D_{\rm w}}{2m\Gamma}, \end{aligned} \tag{52}$$

$\left(q_1^{(\rm n)}(E)\right)^*$ is a complexly conjugated quantity to the first-order Fourier component of coordinate as function of energy and phase for the conservative system with the noise-modified potential $U^{(\rm n)}(q)$,

$$\begin{aligned} &q_1^{(\rm n)}(E) = \frac{1}{2\pi}\int_0^{2\pi} \mathrm{d}\varphi \exp(-\mathrm{i}n\varphi) q^{(\rm n)}(E,\varphi), \\ &\left(q_1^{(\rm n)}(E)\right)^* = \frac{1}{2\pi}\int_0^{2\pi} \mathrm{d}\varphi \exp(\mathrm{i}n\varphi) q^{(\rm n)}(E,\varphi), \end{aligned} \tag{53}$$

and $W_1(E,\Omega)$ is a solution of a boundary problem which reads in a compact form as follows:

$$\begin{aligned} &-\mathrm{i}\left[\Omega-\omega^{(\rm n)}\right]W_1 = 2\Gamma\left\{\left[1+\frac{\overline{\left(p^{(\rm n)}\right)^2}}{m}\frac{\mathrm{d}}{\mathrm{d}E}\right]\left[1+\frac{D_{\rm w}}{2m\Gamma}\frac{\mathrm{d}}{\mathrm{d}E}\right]-\frac{D_{\rm w}}{2m\Gamma}\left(\omega^{(\rm n)}\right)^2\overline{\left(q_E^{(\rm n)}\right)^2}\right\}W_1 + q_1^{(\rm n)}\tilde{W}_{\rm Gibbs}, \\ &W_1(E=0,\Omega)=0, \qquad W_1(E=E_{\rm up},\Omega)=0, \\ &\frac{D_{\rm w}}{2m\Gamma} << \Delta U, \end{aligned} \tag{54}$$

where $\omega^{(\rm n)} \equiv \omega^{(\rm n)}(E)$ is the eigenfrequency vs. energy for the noise-modified dc voltage (see Supplementary Equation 25 in Supplementary Note 2),

$$\omega^{(n)}(E) = \omega(E) - \frac{D_{w} f_{bd} g}{\sqrt{\ln(2)/\pi} m\omega_{s} \varepsilon S V_{dc}^{2}} \left(1 + \frac{3}{m g^{2} \omega_{s}^{2}} E\right)$$

(55)

(in the noise-induced part of $\omega^{(n)}(E)$, terms $\propto E^{k}$ with $k \geq 2$ are negligible in the relevant range of E, which is why they are omitted in Supplementary Equation 55) with the bandwidth f_{bd} equal to 741 Hz for the conventional case (Supplementary Figure 1) and to 298 Hz for the zero-dispersion case, the over-bar $\overline{\ldots}$ in Supplementary Equation 54 means the averaging over the phase i.e.

$$\overline{\left(p^{(n)}\right)^{2}} \equiv \overline{\left(p^{(n)}\right)^{2}}(E) = \frac{1}{2\pi} \int_{0}^{2\pi} d\varphi \left(p^{(n)}(E,\varphi)\right)^{2}, \tag{56}$$

$$\overline{\left(q_{E}^{(n)}\right)^{2}} \equiv \overline{\left(q_{E}^{(n)}\right)^{2}}(E) = \frac{1}{2\pi} \int_{0}^{2\pi} d\varphi \left(\frac{\partial q^{(n)}(E,\varphi)}{\partial E}\right)^{2}, \tag{57}$$

and $\tilde{W}_{Gibbs} \equiv \tilde{W}_{Gibbs}(E)$ is the quasi-stationary distribution given in Supplementary Equation 50.

For our system, the ordinary differential equation in Supplementary Equation 54 can be further simplified. In the relevant range of energy E and noise intensity D, the conservative approximation is very close to the harmonic oscillator in the sense that the relative deviation of the coefficients $\left(\omega^{(n)}(E)\right)^{2}$, $\overline{\left(p^{(n)}\right)^{2}}(E)$, $\overline{\left(q_{E}^{(n)}\right)^{2}}(E)$, $q_{1}^{(n)}(E)$ and $\tilde{W}_{Gibbs}(E)$ from those in the harmonic approximation is negligible (being $\lesssim 10^{-5}$) and therefore all these coefficients in the right-hand side of the differential equation in Supplementary Equation 54 may be replaced by their harmonic approximations. On the contrary, the deviation of $\omega^{(n)}(E)$ from ω_{1} in the imaginary left-hand side of the

equation plays the crucial role: it is just the behavior of this deviation as function of E that determines main features of the fluctuational spectrum. Using the harmonic approximations for the coefficients in the right-hand side of the differential equation in Supplementary Equation 54 and introducing proper normalizations of relevant quantities and parameters, we can present the procedure of finding the fluctuation spectrum in the following rather simple way. The spectrum is equal to

$$\tilde{Q}(\Omega) = \frac{\langle E \rangle}{2m\Gamma\omega_1^2} \int_0^{E_{\mathrm{up}}/\langle E \rangle} \mathrm{d}x \sqrt{x}\, \mathrm{Re}[y(x)], \tag{58}$$

where x is a dimensionless variable having a meaning of energy E normalized by the average energy $\langle E \rangle$ (given in Supplementary Equation 52), and $y(x)$ is a dimensionless complex function being a solution of the following ordinary differential equation

$$x\frac{\mathrm{d}^2 y}{\mathrm{d}x^2} + (1+x)\frac{\mathrm{d}y}{\mathrm{d}x} + \left(1 - \frac{1}{4x} + \mathrm{i}\frac{\Omega - \omega^{(\mathrm{n})}(E = x\langle E \rangle)}{2\Gamma}\right) y = -\frac{\sqrt{x}}{2\pi}\exp(-x), \tag{59}$$

which satisfies the boundary conditions

$$y(x=0) = y\left(x = E_{\mathrm{up}}/\langle E \rangle\right) = 0. \tag{60}$$

Supplementary Note 4. Allan deviation of the oscillator in the zero-dispersion regime

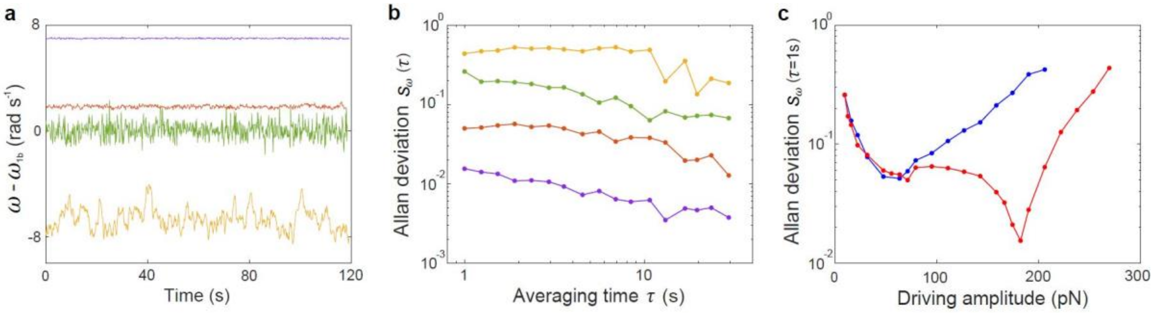

Supplementary Figure 2 Frequency fluctuations and Allan deviation in the zero-dispersion regime. **a** Measured frequency of the self-sustained oscillations driven with feedback for V_{dc} = -1.59V at driving amplitudes of 22.2 pN (green), 71.3 pN (brown), 182 pN (purple) and 269 pN (yellow). **b** Allan deviation of the data in **a** as a function of the averaging time. **c** Allan deviation of the frequency of self-sustained oscillations at the given $\tau = 1$ s versus driving amplitude for oscillators in the conventional regime (blue) and the zero-dispersion regime (red). The Allan deviations are calculated using the same recorded frequencies as those in Fig. 4d of the main text.

In Fig. 4d of the main text, the resonator is driven with feedback using a phase locked loop. The frequency for each data point is recorded over a duration of 120 s. Supplementary Figure 2a shows the recorded frequency as a function of time for four different driving amplitudes of 22.2 pN, 71.3 pN, 182.1 pN and 269 pN. In addition to characterizing the frequency stability using the standard deviation of frequency, we perform the analysis using the Allan deviation:

$$s_\omega(\tau) = \sqrt{\sum_{i=1}^{N-1}(\bar{\omega}_{i+1}^{\tau} - \bar{\omega}_i^{\tau})^2 \Big/ 2(N-1)} \qquad (61)$$

where N is the maximum number of non-overlapping time intervals of duration τ in the total recording time of 120 s. $\bar{\omega}_i^{\tau}$ represents the recorded frequency averaged over the ith

interval. Supplementary Figure 2b shows that $s_\omega(\tau)$ decreases with τ, supporting the notion that the effect of long term frequency drift is small for the recording time. Supplementary Figure 2c plots the Allan deviation s_ω (τ = 1s) as a function of driving amplitude for the zero dispersion and conventional nonlinear regimes, using the same records of frequency as in Fig. 4d in the main text. The improvement of the Allan deviation in the zero dispersion regime over the convention regime is about a factor of 3.6.

Supplementary References

1. Bogolyubov N. N. & Mitropolsky Y. A. *Asymptotic Methods in the Theory of Nonlinear Oscillators* (Gordon and Breach, New York, 1961).
2. Landau L. D. & Lifshitz E. M. *Mechanics* (Elsevier, Amsterdam, 2004).
3. Soskin S. M., Mannella R. & McClintock P. V. E. Zero-dispersion phenomena in oscillatory systems. *Phys. Rep.* **373**, 247-408 (2003).
4. Dykman M. I. & Krivoglaz M. A. Time Correlation-Functions and Spectral Distributions of the Duffing Oscillator in a Random Force-Field. *Physica A* **104**, 495-508 (1980).
5. Risken H. *The Fokker-Planck Equation* (Springer, Berlin, 1992).
6. Landau L. D. & Lifshitz E. M. *Statistical Physics* (Elsevier, London, 1980).
7. Dykman M. I., Mannella R., McClintock P. V. E., Soskin S. M. & Stocks N. G. Noise-Induced Narrowing of Peaks in the Power Spectra of Underdamped Nonlinear Oscillators. *Phys. Rev. A* **42**, 7041-7049 (1990).
8. Dykman M. I.*, et al.* Supernarrow Spectral Peaks and High-Frequency Stochastic Resonance in Systems with Coexisting Periodic Attractors. *Phys. Rev. E* **49**, 1198-1215 (1994).
9. Kramers H. Brownian motion in a field of force and diffusion model of chemical reactions. *Physica* **7**, 284-304 (1940).

10. Feynman R. P. & Hibbs A. R. *Quantum Mechanics and Path Integrals* (McGraw-Hill, New York, 1965).
11. Dykman M. I. Large Fluctuations and Fluctuational Transitions in Systems Driven by Colored Gaussian-Noise - a High-Frequency Noise. *Phys. Rev. A* **42**, 2020-2029 (1990).
12. Soskin S. M. Large fluctuations in multiattractor systems and the generalized Kramers problem. *J. Stat. Phys.* **97**, 609-676 (1999).
13. Aldridge J. S. & Cleland A. N. Noise-enabled precision measurements of a duffing nanomechanical resonator. *Phys. Rev. Lett.* **94**, 156403 (2005).
14. Stambaugh C. & Chan H. B. Noise-activated switching in a driven nonlinear micromechanical oscillator. *Phys. Rev. B* **73**, 172302 (2006).
15. Kaufman I. K., Luchinsky D. G., McClintock P. V. E., Soskin S. M. & Stein N. D. Zero-dispersion stochastic resonance in a model for a superconducting quantum interference device. *Phys. Rev. E* **57**, 78-87 (1998).
